# Protection Conferred by Gallid Alphaherpesvirus 2 Vaccines Against Immunosuppression Induced by Very Virulent Plus (vv+) Marek’s Disease Virus Strains in Commercial Meat Type Chickens

**DOI:** 10.3390/pathogens14010054

**Published:** 2025-01-10

**Authors:** Nagwa Khaled, Carissa Gaghan, Abdelhamid M. Fares, Christa Goodell, William Stanley, Raveendra R. Kulkarni, Isabel M. Gimeno

**Affiliations:** 1 College of Veterinary Medicine, North Carolina State University, Raleigh, NC 27607, USA; nkkhaled@ncsu.edu (N.K.); cegaghan@ncsu.edu (C.G.); amfares@ncsu.edu (A.M.F.); 2 Faculty of Veterinary Medicine, University of Sadat City, El Sadat City 6011007, Menofia Governorate, Egypt; 3 Boehringer Ingelheim Animal Health USA Inc., Duluth, GA 30096, USA; christa.goodell@boehringer-ingelheim.com (C.G.); william.stanley@boehringer-ingelheim.com (W.S.)

**Keywords:** chickens, MDV, immunosuppression, CEO, ILT, CVI-988, rMd5-BAC∆Meq, CVI-LTR

## Abstract

Very virulent plus Marek’s disease virus (vv+MDV) induces severe immunosuppression in commercial chickens. In this study, we evaluated how three Gallid alphaherpesvirus 2 (GaHV-2) vaccines (CVI-988, rMd5-BAC∆Meq, and CVI-LTR) protected against two negative outcomes of vv+MDV infection: (1) reduced viability and frequency of immune cells in the spleen and (2) decreased efficacy of the CEO (chicken embryo origin) vaccine against infectious laryngotracheitis challenge. At 25 days post-infection with vv+MDV 686, all vaccines are protected against the reduced viability of splenocytes. However, there were differences in the frequency of splenic immunophenotypes among groups. Compared to the uninfected control, the frequency of B cells was reduced in the CVI-988/686 group but not in the rMd5-BAC∆Meq/686 and CVI-LTR/686 groups. T cell subset frequencies showed no difference between the negative controls and CVI-988/686; however, there was a reduction in activated CD4+ T cells in the rMd5-BAC∆Meq/686 group and in activated CD4+, activated CD8+, and γδ+ T cells in the CVI-LTR/686 group. We also demonstrated that the three vaccines protected against MDV-induced tumors, but only rMd5-BAC∆Meq and CVI-LTR protected against the negative impact of vv+MDV 648A strain on CEO vaccine efficacy. Our findings demonstrate important differences in the biology and/or mechanisms of protection of these vaccines.

## 1. Introduction

Marek’s disease (MD) is a highly contagious and economically significant disease of chickens caused by Gallid alphaherpesvirus 2 (GaHV-2), which is generally known as Marek’s disease virus (MDV) [1,2]. MD is primarily characterized by the induction of lymphoproliferative tumors and severe immunosuppression in chickens. GaHV-2 belongs to the genus *Mardivirus* and includes all the oncogenic herpesviruses isolated from chickens and their attenuated forms. It was formerly known as serotype 1 or MDV-1. Two other related species are also classified in the *Mardivirus* genus that includes: Gallid alphaherpesvirus 3 or GaHV3 (formerly known as serotype 2 or MDV-2) are non-oncogenic herpesviruses isolated from chickens, and Meleagrid alphaherpesvirus 1 or MeHV1 (formerly known as serotype 3, herpesvirus of turkeys, or HVT) are non-oncogenic herpesviruses isolated from turkeys [1,2,3,4]. In this article, the term MDV refers to the oncogenic GaHV-2 strains. The term MDV-1 vaccine indicates the GaHV-2 vaccine.

MD has been successfully controlled by vaccination since the late 1960s [5,6,7,8]. GaHV-3, HVT, and attenuated strains of MDV-1 are routinely used as vaccines [6,7,9]. Even though vaccines are highly efficacious in preventing the development of tumors, they do not protect against infection or transmission of MDV, thereby contributing to the evolution of MDV toward more virulent strains [10,11]. At least four pathotypes have been recognized based on the ability of MDV strains to break vaccine-induced immunity: mild (m), virulent (v), very virulent (vv), and very virulent plus (vv+) [12,13,14]. vv+MDV strains overcome genetic resistance to the disease and vaccine-induced immunity [15]; they are more neurovirulent [16,17], induce more severe eye lesions [18,19], and they are more immunosuppressive [20,21,22,23]. MDV-induced immunosuppression (MDV-IS) is currently a significant challenge for the poultry industry [24,25]. Novel hypervirulent strains, capable of overpassing immunity conferred by CVI988, have been reported in Asia, showing that the virus is still evolving [26,27,28].

MDV-induced immunosuppression (MDV-IS) is very complex and can occur in chickens that are properly vaccinated and bearing maternal antibodies against MDV in the absence of lymphoid organ atrophy or tumors [29]. It increases the susceptibility of infected birds to other diseases [18] and negatively affects the protection conferred by other vaccines like CEO (chicken embryo origin) against challenges with infectious laryngotracheitis virus (ILTV) [22,30,31]. For decades, it was well recognized that MDV induces two phases of immunosuppression: the early IS phase associated with the early replication of MDV in the lymphoid organs (early-MDV-IS) and the late IS phase associated with the development of tumors (late-MDV-IS) [25]. Because most of the early studies were conducted using vMDV or vvMDV, it was considered that maternal antibodies and/or vaccination prevented all forms of MDV-IS [31]. In recent years, we have demonstrated that vv+MDV could induce a third phase of immunosuppression associated with the reactivation of MDV that was unrelated to the early MDV-IS and the development of tumors [31] We named this phase MDV-IS/R to differentiate it from the late-MDV-IS due to tumors (MDV-IS/T) [22]. We developed a model to reproduce and study MDV-IS/R under laboratory conditions [30]. In this model, MDV-IS/R is indirectly evaluated by assessing the negative effect of early challenge with a vv+MDV strain on the efficacy of a vaccine against infectious laryngotracheitis (ILT) in commercial meat-type chickens. Hence, we refer to it as the “ILT model”. Using this model, it has been demonstrated that MDV-IS/R affects both humoral and cellular immune responses [30], is only induced by vv+MDV [21], is unrelated to lymphoid organ atrophy and tumors, and is not protected by conventional vaccines, including the gold standard CVI-988 vaccine [31]. In our study, only an experimental recombinant vaccine (rMd5-BACΔMeq) [32] based on vvMDV strain Md5 lacking the two copies of the oncogene *meq*, protected against both tumors and MDV-IS/R [31]. rMd5-BACΔMeq is not currently licensed as it induces lymphoid organ atrophy in chickens lacking maternal antibodies against MDV [33]; therefore, it is critical that novel vaccines are developed that can control MDV-IS/R. One potential candidate is the CVI-LTR strain, a novel recombinant MDV vaccine that carries the long terminal repeat (LTR) of the reticuloendotheliosis virus (REV) insertion into the genome of the vaccine CVI-988. This vaccine is licensed and currently being used in various countries [34,35]. Field trials demonstrated that CVI-LTR replicated extensively and earlier than several commercial CVI-988 vaccines in the lymphoid organs and was more successful in reducing oncogenic MDV DNA at 21 days of age than several commercial CVI-988 vaccines [36]. Extensive replication in the lymphoid organs has been associated with higher protection ability against MD [37].

The mechanisms of MDV-IS/R induced by vv+MDV strains are poorly elucidated. In previous studies, we have demonstrated that in meat-type chickens bearing maternal antibodies against MDV, vv+MDV severely decreased the percentage of live cells in the spleen by 20 dpi and significantly decreased the ability of the splenocytes to proliferate when in contact with concanavalin A in vitro [23,38]. More recently, we have further demonstrated that vv+MDV alters the splenic immunophenotypes, inducing a significant decrease in the B cells, CTLs, and γδ+ T cells and upregulating MHC-I expression on T cells at 20 days post-infection (dpi) [23]. The aim of the current study was to evaluate the protection of three MDV-1 vaccines (CVI-988, rMd5-BAC∆Meq, and CVI-LTR) against two negative outcomes of vv+MDV infection: (1) reduced overall splenocyte viability and frequency of immune cells at 6 and 25 dpi, and (2) decreased efficacy of the CEO vaccine against ILT challenge using the ILT model.

## 2. Materials and Methods

### 2.1. Experimental Animals

Commercial-specific pathogen-free SPAFAS chickens (Charles River SPAFAS, N Franklin, CT, USA) were used as MDV shedder chickens as the source of infection in the first experiment. Commercial meat-type chickens bearing maternal antibodies (derived from dams vaccinated with HVT, SB-1, and CVI-988) were used as experimental chickens in the first and second experiments.

### 2.2. Challenge Viruses

Serotype 1 MDV vv+MDV strain 686 at passage 10 in duck embryo fibroblasts and with one additional passage in chicken kidney cells (CKC) [39] was used to challenge the SPAFAS shedder chickens in the first experiment. The vv+MDV strain 648A at passage 10 in DEF [12] was used for the challenge in the second experiment. The use of two different vv+MDV strains was due to logistic issues as we did not have a low passage 686 strain available for the second study; hence, we used a low passage 648A. Previous studies have demonstrated that both 648A and 686 have similar immunosuppressive abilities [21,31,38]. Illinois-N71851 strain of ILTV, which has been characterized as a virulent ILTV strain, was used for the ILT challenge [40,41].

### 2.3. Vaccines

Commercial MD vaccines HVT [6], CVI-988 [7], and CVI-LTR [34] and experimental vaccine rMd5-BACΔMeq [42] were used for protection against MDV-1, and a commercial infectious laryngotracheitis (ILT) chicken embryo origin vaccine (CEO) was used for protection against ILTV.

### 2.4. Experimental Design

Two animal experiments were conducted following the guidance and under the approval of the North Carolina State University Institutional Animal Care & Use Committee (IACUC). In the first experiment, SPAFAS chickens “shedders” were vaccinated in ovo with the HVT vaccine via the amniotic route at 18 embryonic days (ED) to avoid the development of transient paralysis and ensure survival. At hatch, shedder chickens were infected with 500 PFU vv+MDV 686 strain via the subcutaneous route and housed in isolation for 15 days prior to the beginning of the experiment. Female commercial meat-type chickens bearing maternal antibodies were divided into 4 groups (80 chickens per group); 1 was left unvaccinated, and 3 were vaccinated at 18 days of embryonation (ED) with one of the three MDV-1 vaccines CVI-988, rMd5-BAC∆Meq or CVI-LTR. At hatch, chickens were wing banded, and each group was further divided into two groups (40 per group); one was left unchallenged as a control group, and the other group was co-mingled with 15-day-old shedders and challenged by contact. All groups were placed in environment-controlled rooms (BSL-2). Birds from the negative control group and the challenge groups were euthanized at 6 dpi (14 chickens per group) and 25 dpi (7 chickens per group) to collect spleens for flow cytometry. Five chickens from the vaccinated unchallenged groups and from the negative control group were euthanized at 3, 5, and 25 days of age to evaluate vaccine DNA load in the spleen. Five extra chickens were included in each group to account for potential mortality.

The second experiment was conducted using the late-MDV-IS ILT model, as described before [30]. Commercial meat-type chickens were divided into four groups at 18 ED, one was left unvaccinated, and three groups (35 per group) were vaccinated intra-amniotic with one of the three MDV-1 vaccines (CVI-988, rMd5-BAC∆Meq, and CVI-LTR). At hatch, all chickens were wing banded and neck tagged to facilitate monitoring clinical signs and were housed in environment-controlled rooms (BSL-2). The unvaccinated group (120 chickens) was further subdivided into four control groups (30 per group): (1) no treatment; (2) challenged with ILTV; (3) vaccinated with CEO and challenged with ILTV; and (4) infected with 500 PFU of MDV (648A), vaccinated with CEO and challenged with ILTV. The MD-vaccinated groups were challenged with MDV (648A), vaccinated with CEO, and challenged with ILTV. MDV challenges were performed at 4 days of age by the subcutaneous route. CEO vaccine was administered in drinking water at 14 days of age per the manufacturer’s recommendations. ILTV challenge was administered intratracheally at 28 days of age using 2000PFU of a very virulent Illinois ILTV strain. Chickens were individually monitored daily for ILT clinical signs for six days following the ILTV challenge. All chickens were necropsied at termination (day 7 after ILTV challenge) or at the time of death and evaluated for gross lesions consistent with ILT and MD.

### 2.5. Spleen Single-Cell Suspension

Spleen samples (n = 7; two spleens combined per sample at 6 dpi and one spleen per sample at 25 dpi) were obtained in 10 mL of R10 growth medium (RPMI containing 10% FBS, 2.5 mL gentamicin, 5 mL penicillin/streptomycin, and 0.175 mL 2-mercaptoethanol) and kept on ice. The tissues were then homogenized using a plunger from a sterile 5 cc syringe. The resulting cell suspensions were gently passed twice through 40 µm cell strainers (Fisher scientific^®^, Pittsburgh, PA, USA) to isolate individual cells. Following centrifugation at 400× *g* for 10 min at 4 °C, the cell pellet was loosened by gentle flicking. To eliminate red blood cells 1× ACK (Ammonium–Chloride–Potassium) lysis buffer (Biowhittaker^®^, Lonza, VWR Scientific, Mississauga, ON, Canada) was used by adding a volume of 1.5 mL per pellet and incubated at room temperature for 5 min. Finally, splenocytes were resuspended in a 5 mL R10 growth medium. Using a hemocytometer and trypan blue stain, cells were counted, and density was adjusted to a final concentration of 10^7^ cells/mL.

### 2.6. Flow Cytometry-Based Cellular Analysis

Cells were plated in 96 well round-bottom plates, with each well containing 10^6^ cells in 100 μL FACS buffer. Directly conjugated-fluorescent mouse monoclonal antibodies against chicken cell markers (Southern Biotech, Birmingham, AL, USA) were added to each well (0.5–1 μg/10^6^ cells) and stained for 30 min on ice. The cells were stained in different panels of antibody staining due to the paucity of chicken antibody reagents available in multicolor formats (Supplementary Table S1). Cell viability dye, Live/Dead™ near-infrared (Invitrogen, Carlsbad, CA, USA), was used in all panels to exclude dead cells. Cells were washed twice in FACS buffer and centrifuged at 1650× *g* for 5 min at 4 °C. Then the cells were fixed in 4% paraformaldehyde (PFA) for 10 min, centrifuged, resuspended in 200 × PBS, transferred to 5 mL round-bottom polystyrene tubes (BD Falcon 352052, Becton Dickinson, Franklin Lakes, NJ, USA) and kept covered on ice until analysis. Alongside these samples, negative controls (two replicates each), including unstained cells and single-color controls, were used, where they consisted of cells treated with one of the monoclonal antibodies mentioned above so that each antibody was represented individually and served as compensation controls. Each control well received 1 μL of the antibody and 100 μL of FACS buffer. Fluorescence minus one was also used for gating control. Data acquisition was performed using an LSR-II flow cytometer (BD Biosciences, Franklin Lakes, NJ, USA). Subsequent data analysis was performed using Flow Jo software v10 (Tree Starr, Ashland, OR, USA). The gating strategy included the exclusion of doublet cells through forward and side scatter plotting using area (A), height (H), and width (W), followed by gating on live cells and dead cells and further gating of the specific cell population in each panel (Supplementary Figures S1–S4). Frequencies of different cell populations were determined and analyzed using statistical analysis.

### 2.7. DNA Extraction and qPCR

The DNA was extracted from the spleens by using the Gentra Puregene Tissue kit (QIAGEN, Inc., Germantown, MD USA) following the manufacturer’s recommendations. Using a previously described real-time PCR assay [43], the extracted DNA samples were amplified. In brief, each sample underwent amplification using two primer sets targeting the glycoprotein B (*gB*) gene of MDV-1 (amplifies *gB* gene of oncogenic MDV and all vaccines used in this study) and chicken *GAPDH* gene (as a housekeeping gene). The sequences for the respective forward and reverse primers were as follows: TM.5 (5′-CGGTGGCTTTTCTAGGTTCG-3′) and TM.3 (5′-CCAGTGGGTTCAACCGTGA-3′) for MDV-1 gB gene; GAPDH-TM.5 (5′-GGAGTCAACGGATTTGGCC-3′) and GAPDH-TM.3 (5′-TTTGCCAGAGAGGACGGC-3′) for chicken *GAPDH* gene [22]. PCR amplification was conducted using the MX3005P^®^ Stratagene (Stratagene, La Jolla, CA, USA) in a 25 μL reaction containing 50 ng of DNA, 0.2 μM of each primer and BRILLIANT^®^ SYBR^®^ Green QPCR Master Mix (Molecular Probes, Eugene, OR, USA) which contained the appropriate buffers, nucleotides, and Taq-polymerase (Biocrest-Stratagene, Cedar Creek, TX, USA). The reaction was cycled 45 times at 95 °C denaturation for 15 s and a 60 °C combined annealing/extension for 60 s. Fluorescence was acquired at the end of the annealing/extension phase. Melting curves were generated at the end of amplification by cooling the sample at 2 °C/s to 60 °C and then increasing the temperature to 95 °C at 0.1 °C/s. The threshold cycle (Ct) was calculated for each PCR by establishing a fixed threshold. A sample was considered positive if it crossed the threshold line before the end of the reaction (45 cycles). Ct is defined as the fractional cycle number at which the fluorescence surpasses the fixed threshold. The comparative Ct method (DDCT), accounting for primer efficiency, was used to assess the relative quantification of MDV vaccine DNA load, as described by Pfaffl (2001) [44].

### 2.8. Assessment of MDV-Induced Tumors

MDV-induced tumors were evaluated by gross inspection at the end of the experiment or when chickens died. % MD (visceral tumors, enlarged nerves, or skin lesions) was calculated. The efficacy of MD vaccines was calculated as the protection index (PI-MD), with *-/MDV* the unvaccinated and challenged group and *MD-vaccinated/MDV* the vaccinated and challenged groups.

PI-MD = (%MD_-/MDV_ − %MD _MD-vaccinated/MDV_)/%MD_-/MDV_ × 100

### 2.9. Assessment of Late-MDV-IS

Evaluation of late-MDV-IS was performed based on ILT clinical signs (CS) and gross lesions (GL) described in detail by Faiz et al. [30]. For each chicken, the number of days showing CS was recorded and scored (values 0–6). GL in the trachea at termination was scored based on severity (values 0–4). ILT index was calculated to assess the severity of the disease based on CS and GL.

ILT index (ILTI) = CS + GL (values 0–10)

The protection index of the CEO vaccine (PI CEO) was calculated using ILT index values from the positive control group (-/ILTV) and the CEO vaccinated groups (-/CEO/ILTV, and MDV/CEO/ILTV) to measure the protection provided by the CEO vaccine.

PI CEO = (ILTI-/ILTV − ILTITXT)/(ILTI-/ILTV) × 100

ILTI-/ILTV is the ILT index of the -/-/ILTV control group.

ILTI_TXT_ is the ILT index of any treatment group that had been vaccinated with the CEO vaccine.

The results are presented as PI-ILT, which represents PI-CEO in the MDV-challenged groups (MD vaccinated or unvaccinated) relative to the value of the PI-CEO in the control group (None/None/CEO/ILTV).

### 2.10. Statistical Analysis

Data were analyzed using GraphPad Prism v10 (GraphPad software, San Diego, CA, USA). Data were first tested for normality using the Shapiro–Wilk test. Depending on the distribution, one-way ANOVA followed by Fisher’s LSD test for multiple comparisons or the nonparametric test (Kruskal–Wallis) followed by Dunn’s test for multiple comparisons was used to compare between groups. Significance was considered when the value of *p* was less than or equal to 0.05 (*p* ≤ 0.05).

## 3. Results

### 3.1. Effect of vv+MDV 686 Strain on the Percentage of Live Cells in the Spleen at 6 and 25 dpi and Protection Conferred by MDV-1 Vaccines

**Figure 1:**
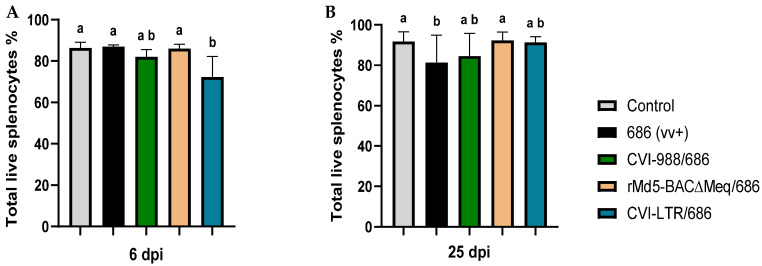
Percentage of live cells in the spleen at 6 and 25 dpi: After 686 infection, single-cell suspensions from spleens of negative control, unvaccinated 686-challenged, and the 3 vaccinated challenged groups were collected at 6 dpi (**A**) and 25 dpi (**B**) for flow cytometry analysis. Cell viability dye, Live/Dead™ near-infrared (Invitrogen, Carlsbad, CA, USA), was used to exclude dead cells. The frequency of live cells was analyzed at each time point using a one-way ANOVA test (parametric or nonparametric). Each data point represents the mean percentage of cells from seven samples of each respective treatment, and error bars represent the standard deviation. Different letters (a, b, ab) above the bars indicate that differences were significant (*p* ≤ 0.05).

The percentage of live cells in the spleen of unvaccinated chickens challenged with vv+MDV strain 686 (686 group) was not affected at 6 dpi (Figure 1A) but was significantly decreased (81.46%) at 25 dpi when compared with the negative controls (91.78%) (Figure 1B). The three evaluated vaccines protected to a certain extent against the negative impact of 686 MDV strain on cell viability at 25 dpi; rMd5-BAC∆Meq vaccine was the most effective (92.35%), followed by CVI-LTR (91.38%), and CVI-988 (84.63%) (Figure 1B). It was noted that at 6 dpi, chickens from the groups CVI-988/686 (numerical) and CVI-LTR/686 (significant) had a lower percentage of live cells (CVI-988/686 = 82.1%, and CVI-LTR/686 = 72.4%) when compared to other groups, including the unvaccinated challenged group (Figure 1A).

### 3.2. Effect of vv+MDV 686 Strain on the Percentage of T Lymphocytes and Macrophages in Spleen at 6 dpi and Protection Conferred by MDV-1 Vaccines

**Figure 2:**
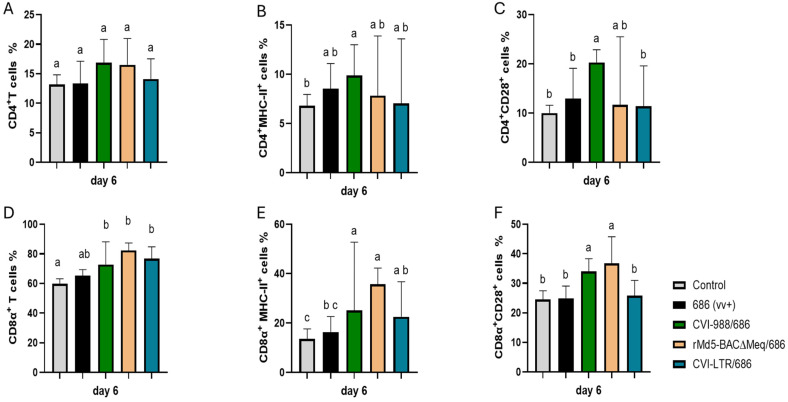
Effect of vv+MDV 686 strain on the percentage of T lymphocytes and their activation in the spleen at 6 dpi and protection conferred by MDV-1 vaccines: At 6 days post-infection (dpi), single cell suspension from spleens of control negative, 686 challenged and vaccinated challenged groups were collected for flow cytometry analysis. Cell viability dye, Live/Dead™ near-infrared (Invitrogen, Carlsbad, CA, USA), was used to exclude dead cells. After gating on live cells followed by CD3+ cells, frequencies of CD4+ (**A**), CD4+ MHC-II+ (**B**), CD4+CD28+ (**C**), CD8α+ (**D**), CD8α+ MHC-II+ (**E**), and CD8α+CD28+ (**F**) T cells were evaluated using one way ANOVA test (parametric or nonparametric). Each data point represents the mean percentage of cells from seven samples (2 birds pooled in each sample) of each respective treatment. Error bars represent standard deviation, and different letters (a, b, c…) above bars indicate differences were significant (*p* ≤ 0.05).

No differences in the percentage of CD4+ T cells were found between the negative control and all treatment groups, including the unvaccinated challenged group (Figure 2A). An increase in the percentage of CD4+ MHC-II+ T cells was detected in all treatment groups when compared to the negative control (6.8%), albeit such an increase was only statistically significant in the group CVI-988/686 (9.9%) (Figure 2B). The percentage of CD4+ CD28+ T cells was only increased in the group CVI-988/686 (20.3%) (Figure 2C).

All vaccinated challenged groups had significant increase in CD8α+ T cells (CVI-988/686 = 72.8%, rMd5-BAC∆Meq/686 = 82.4%, and CVI-LTR/686 = 76.8%) and CD8α+ MHC-II+ T cells (CVI-988/686 = 25.1%, rMd5-BAC∆Meq/686 = 35.7%, and CVI-LTR/686 = 22.1%) when compared with the negative control (59.7% and 13.5%, respectively) (Figure 2D–E). Groups CVI-988/686 (34%) and rMd5-BAC∆Meq/686 (37%) had also a higher percentage of CD8α+CD28+ T cells than all other groups (Figure 2F).

**Figure 3:**
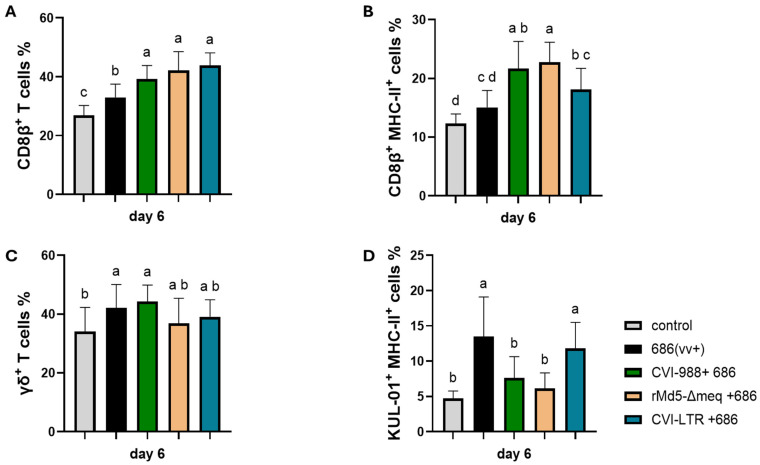
Effect of vv+MDV 686 strain on the percentage of CD8β (CTLs) (**A**) and their activation (**B**), γδ+ T (**C**), and activated macrophages (**D**) in the spleen at 6 dpi and protection conferred by MDV-1 vaccines: At 6 days post-infection, single cell suspension from spleens of negative control, 686 challenged and vaccinated challenged groups were collected for flow cytometry analysis. Cell viability dye, Live/Dead™ near-infrared (Invitrogen, Carlsbad, CA, USA), was used to exclude dead cells. For panels A, B, and C, after gating on live cells, frequencies of CD8β+, CD8β+ MHC-II+, and TCRγδ+ were evaluated within the CD3+ cell population while activated macrophages (KUL-01+ MHC-II+) were evaluated within CD45+ cell population. Data were analyzed using one one-way ANOVA test (parametric or nonparametric); each data point represents the mean percentage of cells from seven samples of each respective treatment. Error bars represent standard deviation, and different letters above bars (a, b, ab…) indicate differences were significant (*p* ≤ 0.05).

Both unvaccinated challenged and vaccinated challenged groups had a significant increase in CD8β+ T cells (cytotoxic T lymphocytes or CTLs) (Figure 3A). However, only the vaccinated challenged groups also observed a significant increase in the percentage of activated CTLs (CD8β+ MHC-II+ T cells); groups CVI-988/686 (21.7%) and rMd5-BAC∆Meq (22.7%) demonstrated the highest percentages (Figure 3B).

The percentage of γδ+ T cells was increased in all treatment groups when compared to the negative control (34%); however, it was only significant in groups unvaccinated 686-challenged (42.1%) and CVI-988/686 (44.3%) (Figure 3C). The percentage of activated macrophages (KUL0-1+ cells) was increased in the unvaccinated 686-challenged (13.5%) and in the CVI-LTR/686 (11.8) groups when compared to the negative control group (4.7%). No difference was found between the negative control group and groups CVI-988/686 (7.7%) or rMd5∆Meq/686 (6.1%) (Figure 3D).

### 3.3. Effect of vv+MDV 686 Strain on the Percentage of Lymphocytes (B and T Cells) and Macrophages in Spleen at 25 dpi and Protection Conferred by MDV-1 Vaccines

**Figure 4:**
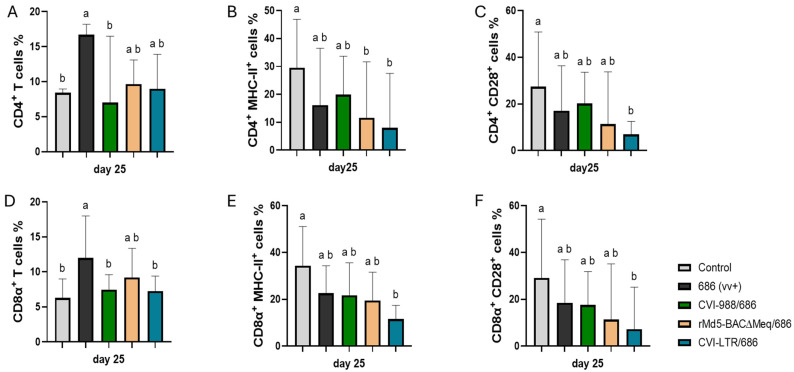
Effect of vv+MDV 686 strain on the percentage of T lymphocytes and their activation in spleen at 25 dpi and protection conferred by MDV-1 vaccines: At 25 days post-infection, single cell suspension from spleens of control negative, 686 challenged and vaccinated challenged groups were collected for flow cytometry analysis. Cell viability dye, Live/Dead™ near-infrared (Invitrogen, Carlsbad, CA, USA), was used to exclude dead cells. After gating on live cells followed by CD3+ cells, frequencies of CD4+ (**A**), CD4+ MHC-II+ (**B**), CD4+CD28+ (**C**), CD8α+ (**D**), CD8α+ MHC-II+ (**E**), and CD8α+CD28+ (**F**) T cells were evaluated and analyzed using one way ANOVA test (parametric or nonparametric). Each data point represents the mean percentage of cells from seven samples of each respective treatment. Error bars represent standard deviation, and different letters above bars indicate differences were significant (*p* ≤ 0.05).

At 25 dpi, the percentage of CD4^+^ T cells was significantly increased in the unvaccinated 686-challenged group (16.7%) compared to the negative control group (8.4%), while no difference was detected in the vaccinated challenged groups (CVI-988/686 = 7%, rMd5-BAC∆Meq/686 = 9.7%, and CVI-LTR/686 = 9%) when compared to the negative control group (Figure 4A). A decrease in the percentage of CD4+ MHC-II+ T cells was detectable in all treatment groups in comparison to the negative control group (29.6%), but this decrease was only significant in the rMd5-BAC∆Meq/686 (11.6%), and CVI-LTR/686 (8%) (Figure 4B). The same trend was found in the percentage of CD4+ CD28+ T cells, but the decrease was only significant in the CVI-LTR/686 group (7%) compared to the negative control group (27.4%) (Figure 4C).

CD8α+ T cells frequency was significantly increased in the unvaccinated 686-challenged group (12%) and numerically in the rMd5-BAC∆Meq/686 (9.2%) while no difference was detected in the CVI-988/686 (7.4%) and CVI-LTR/686 (7.3%) when compared to the negative control group (6.3%) (Figure 4D). The percentage of CD8α+ MHC-II+ T cells was decreased in both unvaccinated challenged and the vaccinated challenged groups (686 = 22.6%, CVI-988/686 = 21.8%, rMd5-BAC∆Meq/686 = 19.5%) but the decrease was only significant in the CVI-LTR/686 (11.6%) when compared to the negative control (34.4%) (Figure 4E). The same trend was found with the percentage of CD8α+ CD28+ T cells. Groups 686-challenged (18.6%), CVI-988/686 (17.5%), rMd5-BAC∆Meq/686 (11.4%) numerically decreased while group CVI-LTR/686 (7.3%) was significantly decreased when compared to the negative control (29.2%) (Figure 4F).

**Figure 5:**
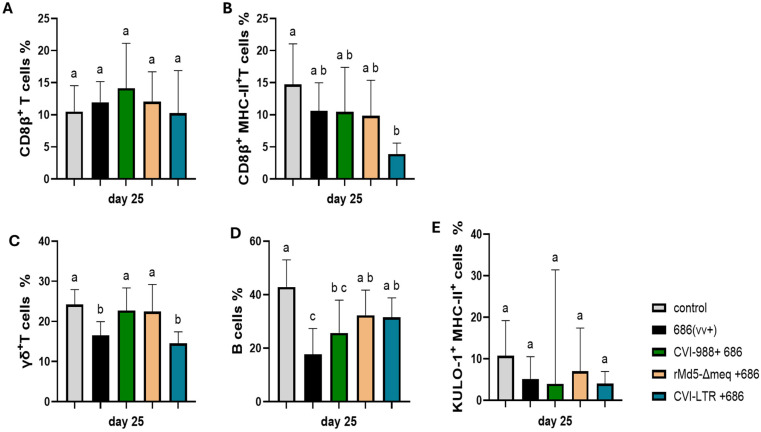
Effect of vv+MDV 686 strain on the percentage of CD8β (CTLs) and their activation, γδ+ T, B cells, and activated macrophages in spleen at 25 dpi and protection conferred by MDV-1 vaccines: At 25 days post-infection, single cell suspension from spleens of control negative, 686 challenged and vaccinated challenged groups were collected for flow cytometry analysis. Cell viability dye, Live/Dead™ near-infrared (Invitrogen, Carlsbad, CA, USA), was used to exclude dead cells. For panel (**A**–**C**), after gating on live cells, frequencies of CD8β+, CD8β+ MHC-II+, and γδ+ T were evaluated within CD3+ cell population while B cells (**D**) and activated macrophages (KUL-01+ MHC-II+) (**E**)) were evaluated within CD45+ cell population. Data were analyzed using a one-way ANOVA test (parametric or nonparametric). Each data point represents the mean percentage of cells from seven samples of each respective treatment. Error bars represent standard deviation, and different letters above bars indicate differences were significant (*p* ≤ 0.05).

No differences in the percentage of CD8β+ T cells were found between the negative control and all treatment groups, including the unvaccinated challenged group (Figure 5A). However, the CD8β+ MHC-II+ T cells percentage was decreased in all treatment groups (686 = 10.6%, CVI-988/686 = 10.5%, rMd5-BAC∆Meq/686 = 9.8%) with significant decrease only in the CVI-LTR/686 group (3.9%), when compared to the negative control (14.7%) (Figure 5B).

The percentage of γδ+ T cells significantly decreased in the unvaccinated 686-challenged group (16.6%) and in the CVI-LTR/686 vaccinated challenged group (14.5%), while no differences were detected in the CVI-988/686 and rMd5-BAC∆Meq/686 groups when compared to the negative control (24.4%) (Figure 5C).

B cell percentage was significantly decreased in the unvaccinated 686-challenged group (8.9%) and in the CVI-988/686 vaccinated challenged group (9.4%) when compared to the negative control (16.6%). No differences in the percentage of B cells were found between the negative control group and rMd5-BAC∆Meq/686 nor CVI-LTR/686 groups (Figure 5D).

The percentage of activated macrophages (KUL0-1+ MHC-II+ cells) was numerically decreased in all the treatment groups including the unvaccinated 686-challenged group (686 = 5.2%, CVI-988/686 = 4%, rMd5-BAC∆Meq/686 = 7.3%, CVI-LTR/686 = 4.1%) when compared to the negative control (10.8%), but no significant differences were detected (Figure 5E).

### 3.4. Effect of vv+MDV 686 Strain on the Expression of MHC-I Expression on Spleen T Cells at 25 dpi and Protection Conferred by MDV-1 Vaccines

**Figure 6:**
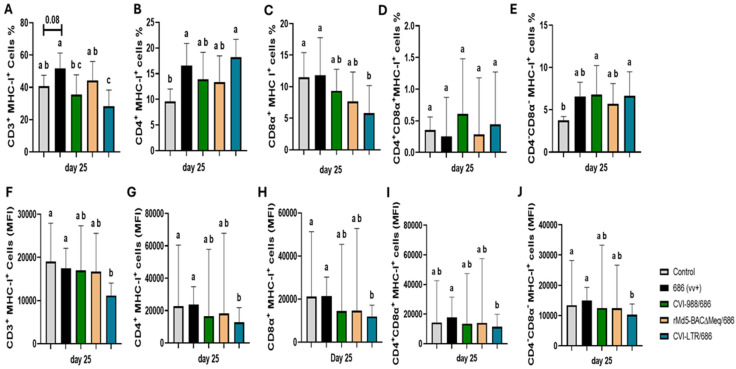
Effect of vv+MDV 686 strain on the MHC-I expression on different T cell subsets in spleen at 25 dpi and protection conferred by MDV-1 vaccines: At 25 days post-infection, single cell suspension from spleens of control negative, 686 challenged and vaccinated challenged groups were collected for flow cytometry analysis. Cell viability dye, Live/Dead™ near-infrared (Invitrogen, Carlsbad, CA, USA), was used to exclude dead cells. After gating on live cells, frequencies and median Fluorescence intensity of MHC-I in CD3+ (**A**,**F**), CD4+ (**B**,**G**), CD8α+ (**C**,**H**), CD4+CD8α+ (**D**,**I**), CD4-CD8α- (**E**,**J**) were evaluated and analyzed using one way ANOVA test (parametric or nonparametric). Each data point represents the mean percentage of cells from seven samples of each respective treatment. Error bars represent standard deviation and different letters above bars indicate differences were significant (*p* ≤ 0.05).

MHC-I expression (percentage of cells expressing MHC-I and median fluorescence intensity, MFI) was evaluated at 25 dpi on different T cell subsets (Figure 6). Compared to the negative control (40.8%), the percentage of CD3+MHC-I+ T cells was increased in the unvaccinated 686-challenged group (51.6%), albeit not significantly (*p* = 0.08). However, it was significantly decreased in the CVI-LTR/686 group (28.3%) and unaffected in groups CVI-988/686 (35.4%) and rMd5-BAC∆Meq/686 (44.3%) (Figure 6A). A similar trend was observed when evaluating MFI. There was a significant reduction in MHC-I intensity in the CVI-LTR/686 group (11,179.3) when compared with the negative control (19,061), while no difference was found in the other treatment groups, including the unvaccinated 686-challenged (Figure 6F).

The percentage of CD4+ MHC-I+ T cells was significantly increased in the unvaccinated 686-challenged (16.6%) and CVI-LTR/686 (18.2%) groups compared to negative control group (9.6%), but no difference was detected in CVI-988/686 (13.9%) and rMd5-BAC∆Meq/686 (13.3%) groups (Figure 6B). In contrast, MFI analysis showed a significant decrease in MHC-I intensity on CD4+ T cells in the CVI-LTR/686 group (12,832) and no differences between other treatment groups and the negative control (22,793) (Figure 6G).

The percentage of CD8α+ MHC-I+ T cells was decreased in all the vaccinated challenged groups, but this decrease was only significant in the CVI-LTR/686 group (5.8% vs. 11.5% in negative control). No difference was detected in the unvaccinated 686-challenged group when compared to the negative control (Figure 6C). The same trend was observed when MFI was evaluated (Figure 6H).

No differences in the percentage of CD4+CD8α+ MHC-I+ T cells nor the intensity of MHC-I expression on CD4+CD8α+T cells were detected among groups (Figure 6D,I). However, the percentage of CD4-CD8- MHC-I+ T cells was increased in all treatment groups, including the unvaccinated 686-challenged group compared to the negative control (3.7%), and such differences were significant in groups CVI-988/686 (6.8%) and CVI-LTR/686 (6.7%) (Figure 6E). By contrast, MFI evaluation revealed a significant reduction in MHC-I intensity in the CVI-LTR/686 group (10,252) compared to the negative control group (13,428.5) and no difference between all other treatment groups and the negative control (Figure 6J).

### 3.5. Vaccine Replication in the Spleen

To monitor the vaccine replication rate, spleen samples were collected from vaccinated non-challenged groups at 3, 5, and 26 days of age. The three vaccines were readily detected at 3 days, and no differences were observed between them. At 5 days, the DNA load of the CVI-LTR vaccine (mean = 588.6) was numerically lower than the DNA load of CVI-988 (mean = 915.4) and rMd5-BAC∆Meq (mean = 1108), although no significant difference was found among vaccinated groups. Both rMd5-BAC∆Meq (*p* = 0.08) and CVI-LTR (*p* < 0.05) were detected in fewer chickens and at lower levels than CVI-988 at 25 days (Supplementary Figure S5).

### 3.6. Ability of MDV-1 Vaccines to Protect Against Late MDV IS and the vv+ 648A Impact on the Efficacy of CEO Vaccine

**Figure 7:**
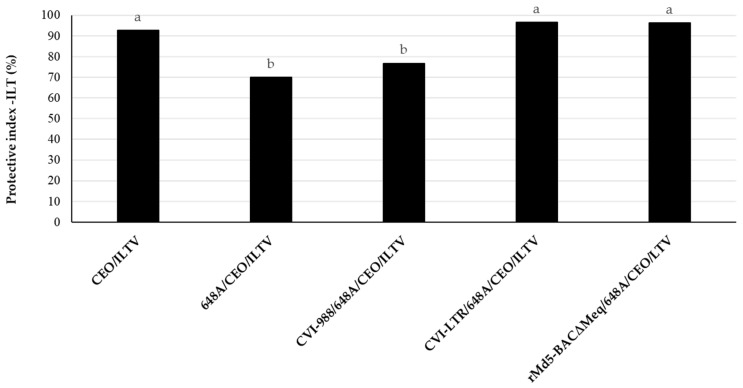
Ability of MDV-1 vaccines, highly protective against MDV-induced tumors, to protect against late-MDV-IS. The efficacy of CEO vaccine against challenge with ILTV (PI of CEO) was evaluated in groups that were either unvaccinated or vaccinated against MD with CVI-988, rMd5-BACΔMEQ or CVI-LTR at 18 days of embryonation, challenged with vv+MDV strain 648A at 4 days of age, vaccinated with CEO in water at 15 days of age, and challenged with ILTV at 30 days of age. LT clinical signs were recorded individually daily for 6 days after the ILTV challenge. Necropsies of all chickens were conducted at the time of death or at 7 days post ILTV challenge. The ILT index was calculated based on the clinical signs and gross lesions, as indicated in Materials and Methods. The results are presented as the protective index of CEO against LT (PI-ILT) in each treatment group. Different letters on the bars indicate that differences were statistically significant (*p* < 0.05).

Challenge with vv+MDV 648A strain at 4 days of age by s/c injection (group 648A/CEO/ILTV) resulted in a significant reduction (PI value of 70) in the CEO vaccine efficacy when compared to the control group CEO/LTV (PI value of 93). Vaccination with CVI-LTR and rMd5-BAC∆Meq (both PI values of 96) significantly protected against the reduction in CEO protection, and they were both similar to the control group (CEO/ILTV). In contrast, vaccination with CVI-988 (CVI-988/648A/CEO/ILTV) did not protect (PI value of 77) against the negative effect of 648A on the protection conferred by CEO, and it was significantly lower than the control group (CEO/ILTV) (Figure 7).

### 3.7. Vaccine Protection Against MDV-Induced Tumors

**Figure 8:**
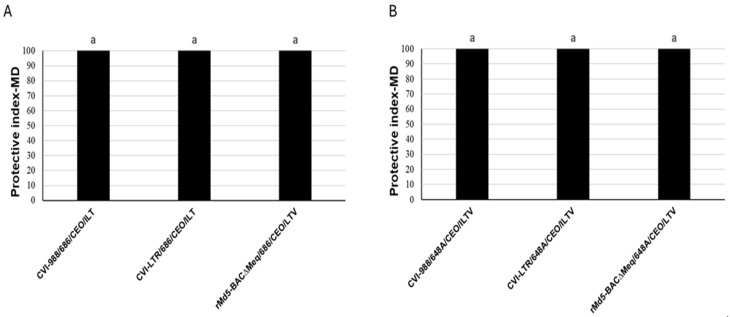
Protection of MDV-1 vaccines against MDV-induced tumors in experiment 1 (**A**) and 2 (**B**): Efficacy of vaccine protection against MD tumors was calculated as the protection index: PIMD = (%MDpositive control − %MDvaccinated group/%MDpositive control × 100. The same letter above the columns indicates that there were no significant differences when *p* ≤ 0.05).

The three evaluated vaccines protected against the development of tumors in the two experiments (PI = 100) (Figure 8). The unvaccinated challenged group developed a high percentage of tumors. In experiment 1, 85% of the positive control group (unvaccinated and challenged with the 686 strain at day of age by contact) developed tumors. In the second experiment, 65% of the positive control group (unvaccinated and challenged with the 648A strain at 4 days via S/C) developed MD lesions.

## 4. Discussion

The present study provides valuable mechanistic insights into the protection conferred by three MDV-1 vaccines against early challenge with the vv+MDV 686 strain. We have previously reported that vv+MDV strains induce severe IS in meat-type commercial chickens [21,22,38] splenic immunophenotypes [23,38], thus jeopardizing protection conferred by other vaccines (for example, ILT vaccines) [30]. In the present study, we have demonstrated that the three most protective MDV-1 vaccines (two commercially available and one experimental) induced strong activation of cytotoxic T lymphocytes (CTLs) at 6 dpi and conferred very good protection against MDV-induced tumors. They also could rescue splenic immune cell death at 25 dpi. However, there were statistically significant differences among treatment groups in the frequency of splenic immunophenotypes and in their ability to protect against the negative impact of vv+MDV 648A strain on CEO vaccine efficacy, suggesting subtle but significant differences in vaccines’ biology and/or mechanisms of protection. Compared to the uninfected control, the frequency of B cells was reduced in the CVI-988/686 group but not in the rMd5-BAC∆Meq/686 and CVI-LTR/686 groups. T cell subset frequencies showed no difference between the negative controls and CVI-988/686; however, there was a reduction in activated CD4+ T cells in the rMd5-BAC∆Meq/686 group and in activated CD4+, activated CD8+, and γδ+ T cells in the CVI-LTR/686 group. Furthermore, only rMd5-BAC∆Meq and CVI-LTR protected against the negative impact of the vv+MDV 648A strain on CEO vaccine efficacy. These findings contribute to our understanding of MD biology and vaccine-induced immunity.

It is well established that cell-mediated immunity is the basis for protection against MD, and CD8+ T cell immune responses elicited by vaccination correlate with reduced viral replication and enhanced protection [45,46,47]. Gimeno et al., 2004 demonstrated that highly protective MDV-1 vaccines could induce activation and expansion of CD4+ helper T cells and CD8+ cytotoxic T cells at 4 days post-vaccination in 15 × 7 chickens lacking maternal antibodies against MD [37]. In commercial meat-type chickens bearing maternal antibodies, Islam et al. showed that CVI-988 requires 5–10 days to induce cell-mediated immunity [48]. In the current study, we found that the three evaluated vaccines (CVI-988, rMd5-BACΔMeq, and CVI-LTR) elicited strong activation of CD8+ T cells (both CD8α+ and CD8β+) at 6 dpi in commercial meat-type chickens. The frequency of CTLs at 25 dpi, however, differed greatly among groups. No difference among treatment groups, including the unvaccinated 686-challenged group, was found in the frequency of CD8β+ T cells (CTLs). In a previous study, we reported that 686 decreased the frequency of CD8β+ T cells at 20 dpi but not at 30 dpi [23]. Since the genetics of the chickens and the virus strain used in the two studies were the same, it is possible that the effect of 686 on CTLs is transient and happens at 20 dpi or earlier but was not detectable at 25 nor 30 dpi. Interestingly, the CVI-LTR/686 group, but none of the other treatment groups, had reduced activated CTLs (CD8β+ MHC-II+ T cells). If the reduction in activated CTLs in this group is due to cell migration out of the spleen, reduced activation of the CTLs or lysis of activated CTLs needs further evaluation.

Previous research has shown that the primary target cells for latency and transformation of MDV are activated CD4+ T cells [49,50,51]. However, if CD4+ T cells are depleted, the virus can also infect and transform other T cell subgroups [50,51,52]. In the present study, we found differences in the frequency of activated CD4+ T cells among treatment groups at both 6 and 25 dpi. At 6 dpi, only CVI-988/686 had increased frequencies of CD4+ MHC-II+ and CD4+ CD28+ T cells. Because activation of CD4+ T helper cells is necessary to activate CTLs [53], and all three vaccinated challenged groups had expansion and activation of CTLs at 6 dpi, it is likely that activation of helper T cells might have occurred in groups rMd5-BACΔMeq/686 and CVI-LTR/686 at an earlier time [37]. The expansion of CD4+ T cells at 25 dpi in the 686 group strongly suggests the development of tumors since CD4+ T cells are the target for MDV transformation. This is supported by the fact that the three vaccines that protected against the development of tumors also protected against the expansion of CD4+ T cells at 25 dpi. The frequency of activated CD4+ T cells, however, varied between treatment groups. While there was no difference between CVI-988/686 and the negative control groups, the frequency of activated CD4+ T cells was significantly decreased in the rMd5-BACΔMeq/686 and CVI-LTR/686 groups.

Recently, the role of γδ+ T cells in MDV pathogenesis has been highlighted by several studies [54,55,56,57]. Hao et al. reported that the CVI-988 vaccine, but not vvMDV RB1B strain infection, induced the expansion of γδ+ T cells and concluded that they were an important part of the CVI-988-induced immune response [56]. Sabsabi et al., using γδ+ T knockout chickens, demonstrated that the absence of γδ+ T cells in MDV-infected chickens resulted in increased viral load in spleen and thymus as well as increased tumor incidence, suggesting an antiviral role early in the infection [57] However, Matsuyama et al., detected two distinct functional subsets of chicken γδ+ T cells (IFN-γ+ and TGF-β+). These authors suggested that IFN-γ producing γδ+ T cells exerts a cytotoxic immune response while the TGF-β γδ+ T cells might play a role as a regulatory T cell subset in MDV infection [54]. In our study, we detected an increase in γδ+ T cells in both CVI-988/686 and unvaccinated 686-challenged groups at 6 dpi. Unlike Hao et al. [56], we cannot conclude that the increase in γδ+ T cell percentage is vaccine-specific because the increase also occurred in the unvaccinated 686-infected group. Differences in the pathotypes used in our study (686 is a vv+MDV while RB1B is a vvMDV) or in the chicken genetics might have contributed to such differences. Because we did not characterize the different subsets of γδ+ T cells in our study, it is unknown if the increased γδ+ T cells detected at 6 dpi in unvaccinated 686-challenged, and CVI-988/686 groups belong to the same phenotype; thus, further studies are warranted. We could detect an increase in the frequency of γδ+ T cells neither in rMd5-BACΔMeq/686 nor in CVI-LTR/686 groups at 6 dpi. However, it is possible that an increase in γδ+ T cells might have occurred at an earlier time point in those two groups as both vaccines rMd5-BACΔMeq and CVI-LTR replicates in the lymphoid organs earlier than CVI-988 [33,36].

Another important innate immune cell type is macrophages, which mediate antiviral responses through secreted cytokines and/or nitric oxide [58]. However, macrophages can play a dual role in MDV pathogenesis as vv+MDV strains can actively replicate in them [59,60,61,62]. MDV has also been found to modulate the secretion of migration inhibitory factor (MIF) that affects the migration of activated macrophages and enhances their survival by suppressing apoptosis [63,64]. In the present study, we detected an increase in the percentage of activated macrophages in the CVI-LTR/686 group and unvaccinated 686-challenged group at 6 dpi but not in rMd5-BAC∆Meq/686 or CVI-988/686 group. Further studies are warranted to elucidate if there are differences in the macrophage phenotypes within these two groups and the significance of this increase in the pathogenesis of 686.

The CVI-LTR/686 group had a reduced percentage of live cells in the spleen at 6 dpi, unlike all other treatment groups, including the unvaccinated 686-challenged group. None of the cell phenotypes evaluated in this study (T cells and macrophages) were reduced in this group. It is possible that other cell populations (i.e., B cells, ellipsoid cells, dendritic cells, and others) are affected, and further studies will be needed to elucidate which cell subtype is reduced in this group and the relevance of such decrease in the vaccine-induced protection. As we described in previous work [23], 686 reduced the percentage of live cells in the spleen at 25 dpi, mainly due to decreased B cells and γδ+ T cells. The three evaluated vaccines protected against the reduced percentage of live cells, although differences among vaccines were observed. CVI-988 vaccine protected against γδ+ T cell reduction but was less effective in preventing the severe depletion of B cells. The CVI-LTR vaccine, on the other hand, provided protection against the loss of B cells but did not counteract the reduction in γδ+ T cells. The rMd5-BAC∆Meq vaccine provided full protection against both B cells and γδ+ T cells.

Downregulation of MHC-I is one of the mechanisms that MDV uses to evade the cell-mediated immune response [65]. In vitro, MDV down-regulates MHC-I in infected cells but upregulates MHC-I in surrounding non-infected cells [66]. In a recent study, it was demonstrated that MDV downregulated MHC molecule BF2 while it prevented the downregulation of MHC molecule BF1, which interacts specifically with NK cells, thereby inhibiting NK-cell activation [67]. In a previous study, we reported that vv+MDV strain 686 increased the frequency of T cells expressing MHC-I [23]. Because the MHC-I monoclonal antibody (F21-2) cannot distinguish between the intact MHC-I with peptide epitope and the denatured MHC-I, our results were inconclusive. In this study, we confirmed our previous results, but this time, we used the MHC-I monoclonal antibody C6B12 (Iowa State University), which detects the intact MHC-I molecule, confirming that vv+MDV 686 indeed increased the frequency of CD3+ and CD4+ T cells expressing MHC-I at 25 dpi. The effect of vaccination on the frequency of T cells expressing MHC-I differs with the vaccine used; CVI-988 and rMd5-BAC∆Meq were able to overcome the 686 ability to upregulate MHC-I expression on T cells, while CVI-LTR resulted in overall downregulation of MHC-I on CD3+ and CD8α+ T cells. Interestingly, in the CVI-LTR/686 group, there were discrepancies between the frequency of CD4+ and CD4−CD8− T cells expressing MHC-I and the mean fluorescence intensity (MFI) on those cell subtypes. Such discrepancies could be explained by 1) the vaccine-induced apoptosis of these cells because they are infected with or transformed by MDV, or 2) the vaccine modulated the immune response by downregulating MHC-I on these cells, rendering them susceptible to NK cell-mediated clearance. Further research is warranted to elucidate the mechanisms by which CVI-LTR modulates MHC-I expression and whether this is related to NK cell activation.

In previous studies, we demonstrated that in addition to immunosuppression associated with early replication of the virus in lymphoid organs (early MDV-IS) and immunosuppression associated with the development of lymphomas (MDV-IS/T), vv+MDV strains induced a type of immunosuppression unrelated to early MDV-IS or MDV-IS/T, associated with the reactivation of the virus (MDV-IS/R) [21,22]. We developed a model to reproduce late MDV-IS-R under laboratory conditions (ILT model) in which we indirectly evaluated the ability of vv+MDV strains to reduce the efficacy of ILT vaccine (CEO) against a challenge with a virulent ILTV [30]. In this model, we mimicked field conditions and practices as much as feasible; hence, we used commercial meat-type chickens and administered the CEO vaccine via drinking water, as it is a common practice in the broiler chicken industry. Using this model, we demonstrated that late-MDV-IS could occur in commercial chickens bearing maternal antibodies and properly protected by vaccination against early MDV-IS, tumors, and MDV-IS/T. None of the conventional MD vaccines (including the gold standard CVI-988) could protect against MDV-IS/R. Only the experimental vaccine rMd5-BAC∆Meq showed the ability to protect efficiently against both tumors and MDV-IS/R [31]. In the present study, we confirmed our previous results and demonstrated that the CVI-LTR vaccine can also protect against MDV-IS/R. This finding is of great relevance as CVI-LTR, but not rMd5-BAC∆Meq, is commercially available. In addition, identifying features common to these two vaccines that differ from those found in CVI-988 will be critical to understanding mechanisms of protection against MDV-IS/R. In the current study, we observed that both CVI-LTR and rMd5-BAC∆Meq replicated early in the lymphoid organs, with detectable levels at 3 days of age and lower DNA load in the spleen at 26 days of age compared to CVI-988. Additionally, both vaccines protected against severe depletion of B cells induced by the 686 at 25 dpi, while CVI-988 was less effective in this regard. Also, both vaccines reduced the percentage of activated T cells in the spleen to 25 dpi. These similarities suggest that CVI-LTR and rMd5-BAC∆Meq may share common mechanisms that contribute to their efficacy in protecting against various aspects of MDV infection and differentiate them from CVI-988.

## 5. Conclusions

In this study, we compared the efficacy of the three most protective MDV-1 vaccines currently available [7,32,34] against an early challenge with a vv+MDV strain (686 or 648A). As expected, the three vaccines replicated early in the lymphoid organs, elicited a strong CTL response in the spleen at 6 dpi and were very efficacious against the development of tumors. They also protected against the severe cell death caused by 686 in the spleen at 25 dpi. However, only rMd5-BAC∆Meq and CVI-LTR are controlled against MDV-IS/R, which can cause severe losses to the poultry industry. In this study, we identified several features unique to these two vaccines that might contribute to the protection against MDV-IS/R and warrant further studies. In particular, the significance of maintaining a high frequency of B cells at 25 dpi and having a reduced percentage of activated CD4+ T cells at both 6 and 25 dpi needs to be further evaluated. In addition, this study raises more questions about the role of γδ+ T cells and the activation of macrophages in the pathogenesis of vv+MDV strains and in vaccine-induced protection.

## Data Availability

Research data are available upon request from Isabel M. Gimeno.

## Supplementary Materials

The following supporting information can be downloaded at: https://www.mdpi.com/article/10.3390/pathogens14010054/s1, Figure S1: Gating strategy for T cell phenotypes; Figure S2: Gating strategy for B cells; Figure S3: Gating strategy for activated macrophages; Figure S4: Gating strategy used for MHC-I expression on different T cell subset; Figure S5: Replication of vaccines; Table S1: Staining panels for flow cytometry analysis of day 6* and 25 of age in meat type chickens.

## References

[1] Biggs P.M., Purchase H.G., Bee B.R., Dalttin P.J. (1965). Preliminary Report on Acute Marek’s Disease (Fowl Paralysis) in Great Britain. Vet. Rec..

[2] Gatherer D., Depledge D.P., Hartley C.A., Szpara M.L., Vaz P.K., Benkő M., Brandt C.R., Bryant N.A., Dastjerdi A., Doszpoly A. (2021). ICTV Virus Taxonomy Profile: Herpesviridae 2021. J. Gen. Virol..

[3] Marek J. (1907). Multiple Nervenentzündung (Polyneuritis) Bei Hühnern. Deut. Tierarztl. Woch..

[4] Churchill A.E., Biggs P.M. (1967). Agent of Marek’s Disease in Tissue Culture. Nature.

[5] Churchill A.E., Payne L.N., Chubb R.C. (1969). Immunization Against Marek’s Disease Using a Live Attenuated Virus.

[6] Witter R.L., Nazerian K., Purchase H.G., Burgoyne G.H. (1970). Isolation from Turkeys of a Cell-Associated Herpesvirus Antigenically Related to Marek’s Disease Virus. Am. J. Vet. Res..

[7] Rispens B.H., van Vloten H., Mastenbroek N., Maas H.J., Schat K.A. (1972). Control of Marek’s Disease in the Netherlands. I. Isolation of an Avirulent Marek’s Disease Virus (Strain CVI 988) and Its Use in Laboratory Vaccination Trials. Avian Dis..

[8] Calnek B.W., Schat K.A., Peckham M.C., Fabricant J. (1983). Field Trials with a Bivalent Vaccine (HVT and SB-1) against Marek’s Disease. Avian Dis..

[9] Schat K.A., Calnek B.W. (1978). Protection against Marek’s Disease-Derived Tumor Transplants by the Nononcogenic SB-1 Strain of Marek’s Disease Virus. Infect. Immun..

[10] Dunn J.R., Black Pyrkosz A., Cheng H.H. Pathotyping of Current Marek’s Disease Virus Field Strains and Identification of Sequence Variants to Predict Virulence. Proceedings of the 11th International Symposium on Marek’s Disease and Avian Herpesvirus.

[11] Calnek B.W., Adldinger H.K., Kahn D.E. (1970). Feather Follicle Epithelium: A Source of Enveloped and Infectious Cell-Free Herpesvirus from Marek’s Disease. Avian Dis..

[12] Witter R.L. (1997). Increased Virulence of Marek’s Disease Virus Field Isolates. Avian Dis..

[13] Witter R.L., Calnek B.W., Buscaglia C., Gimeno I.M., Schat K.A. (2005). Classification of Marek’s Disease Viruses According to Pathotype: Philosophy and Methodology. Avian Pathol..

[14] Read A.F., Baigent S.J., Powers C., Kgosana L.B., Blackwell L., Smith L.P., Kennedy D.A., Walkden-Brown S.W., Nair V.K. (2015). Imperfect Vaccination Can Enhance the Transmission of Highly Virulent Pathogens. PLoS Biol..

[15] Bacon L.D., Hunt H.D., Cheng H.H. (2001). Genetic Resistance to Marek’s Disease. Marek’s Dis..

[16] Gimeno I.M., Witter R.L., Reed W.M. (1999). Four Distinct Neurologic Syndromes in Marek’s Disease: Effect of Viral Strain and Pathotype. Avian Dis..

[17] Gimeno I.M., Witter R.L., Neumann U. (2002). Neuropathotyping: A New System to Classify Marek’s Disease Virus. Avian Dis..

[18] Baigent S.J., Davison F., Davison F., Nair V. (2004). Marek’s Disease Virus: Biology and Life Cycle. Marek’s Disease.

[19] Witter R.L. (1983). Characteristics of Marek’s Disease Viruses Isolated from Vaccinated Commercial Chicken Flocks: Association of Viral Pathotype with Lymphoma Frequency. Avian Dis..

[20] Calnek B.W., Harris R.W., Buscaglia C., Schat K.A., Lucio B. (1998). Relationship between the Immunosuppressive Potential and the Pathotype of Marek’s Disease Virus Isolates. Avian Dis..

[21] Faiz N.M., Cortes A.L., Guy J.S., Fletcher O.J., Cimino T., Gimeno I.M. (2017). Evaluation of Factors Influencing the Development of Late Marek’s Disease Virus-Induced Immunosuppression: Virus Pathotype and Host Sex. Avian Pathol..

[22] Faiz N.M., Cortes A.L., Guy J.S., Reddy S.M., Gimeno I.M. (2018). Differential Attenuation of Marek’s Disease Virus-Induced Tumours and Late-Marek’s Disease Virus-Induced Immunosuppression. J. Gen. Virol..

[23] Khaled N., Kulkarni R.R., Käser T., Gimeno I.M. (2024). Temporal Changes in Splenic Immune Cell Populations Following Infection with a Very Virulent plus MDV in Commercial Meat-Type Chickens. Viruses.

[24] Payne L.N., Davison G., Nair V. (2004). Pathological Responses to Infection. Marek’s Disease.

[25] Gimeno I.M., Pandiri A.R., Gimeno I.M. (2013). Virus-Induced Immunosuppression: Marek’s Disease Virus Infection and Associated Syndromes. Immunosuppresive Diseases of Poultry.

[26] Zheng L.-P., Teng M., Li G.-X., Zhang W.-K., Wang W.-D., Liu J.-L., Li L.-Y., Yao Y., Nair V., Luo J. (2022). Current Epidemiology and Co-Infections of Avian Immunosuppressive and Neoplastic Diseases in Chicken Flocks in Central China. Viruses.

[27] Teng M., Zheng L.-P., Li H.-Z., Ma S.-M., Zhu Z.-J., Chai S.-J., Yao Y., Nair V., Zhang G.-P., Luo J. (2022). Pathogenicity and Pathotype Analysis of Henan Isolates of Marek’s Disease Virus Reveal Long-Term Circulation of Highly Virulent MDV Variant in China. Viruses.

[28] Liu J.-L., Teng M., Zheng L.-P., Zhu F.-X., Ma S.-X., Li L.-Y., Zhang Z.-H., Chai S.-J., Yao Y., Luo J. (2023). Emerging Hypervirulent Marek’s Disease Virus Variants Significantly Overcome Protection Conferred by Commercial Vaccines. Viruses.

[29] Gimeno I.M., Schat K.A. (2018). Virus-Induced Immunosuppression in Chickens. Avian Dis..

[30] Faiz N.M., Cortes A.L., Guy J.S., Fletcher O.J., West M., Montiel E., Gimeno I.M. (2016). Early Infection with Marek’s Disease Virus Can Jeopardize Protection Conferred by Laryngotracheitis Vaccines: A Method to Study MDV-Induced Immunosuppression. Avian Pathol..

[31] Faiz N.M., Cortes A.L., Guy J.S., Fogle J.E., Gimeno I.M. (2016). Efficacy of Various Marek’s Disease Vaccines Protocols for Prevention of Marek’s Disease Virus-Induced Immunosuppression. Vaccine.

[32] Lee L.F., Kreager K.S., Arango J., Paraguassu A., Beckman B., Zhang H., Fadly A., Lupiani B., Reddy S.M. (2010). Comparative Evaluation of Vaccine Efficacy of Recombinant Marek’s Disease Virus Vaccine Lacking *Meq* Oncogene in Commercial Chickens. Vaccine.

[33] Dunn J.R., Silva R.F. (2012). Ability of *MEQ*-Deleted MDV Vaccine Candidates to Adversely Affect Lymphoid Organs and Chicken Weight Gain. Avian Dis..

[34] Lupiani B., Lee L.F., Kreager K.S., Witter R.L., Reddy S.M. (2013). Insertion of Reticuloendotheliosis Virus Long Terminal Repeat into the Genome of CVI988 Strain of Marek’s Disease Virus Results in Enhanced Growth and Protection. Avian Dis..

[35] Pritchard J., Mebatsion T., Bublot M. (2022). Modified Marek’s Disease Virus, and Vaccines Made Therefrom. U.S. Patent.

[36] Faiz N.M., Cortes A.L., Phang Y., Gimeno I.M. (2024). Optimizing Protocols for Monitoring in Vivo Replication of a Novel Chimeric Marek’s Disease Vaccine with an Insertion of the Long Terminal Repeat of Reticuloendotheliosis Virus in the CVI988 Strain Genome (CVI-LTR). Avian Pathol..

[37] Gimeno I.M., Witter R.L., Hunt H.D., Reddy S.M., Reed W.M. (2004). Biocharacteristics Shared by Highly Protective Vaccines against Marek’s Disease. Avian Pathol..

[38] Gimeno I.M., Cortes A.L., Reddy S.M., López de Juan Abad B., Käser T., Limsatanun A. (2019). Highly Virulent Marek’s Disease Virus Strains Affect T Lymphocyte Function and Viability of Splenocytes in Commercial Meat-Type Chickens. Avian Pathol..

[39] Witter R.L., Kreager K.S. (2004). Serotype 1 Viruses Modified by Backpassage or Insertional Mutagenesis: Approaching the Threshold of Vaccine Efficacy in Marek’s Disease. Avian Dis..

[40] Marty E.W., Winans R.E. (1971). Immunizing Characteristics of a Tissue-Culture-Origin Modified Live-Virus Ocular Vaccine for Infectious Laryngotracheitis. Avian Dis..

[41] Guy J.S., Barnes H.J., Munger L.L., Rose L. (1989). Restriction Endonuclease Analysis of Infectious Laryngotracheitis Viruses: Comparison of Modified-Live Vaccine Viruses and North Carolina Field Isolates. Avian Dis..

[42] Silva R.F., Dunn J.R., Cheng H.H., Niikura M. (2010). A *MEQ*-Deleted Marek’s Disease Virus Cloned as a Bacterial Artificial Chromosome Is a Highly Efficacious Vaccine. Avian Dis..

[43] Gimeno I.M., Cortes A.L., Silva R.F. (2008). Load of Challenge Marek’s Disease Virus DNA in Blood as a Criterion for Early Diagnosis of Marek’s Disease Tumors. Avian Dis..

[44] Pfaffl M.W. (2001). A New Mathematical Model for Relative Quantification in Real-Time RT–PCR. Nucleic Acids Res..

[45] Morimura T., Cho K.O., Kudo Y., Hiramoto Y., Ohashi K., Hattori M., Sugimoto C., Onuma M. (1999). Anti-Viral and Anti-Tumor Effects Induced by an Attenuated Marek’s Disease Virus in CD4-or CD8-Deficient Chickens. Arch. Virol..

[46] Schat K.A. (1987). Marek’s Disease: A Model for Protection against Herpesvirus-Induced Tumours. Cancer Surv..

[47] Morimura T., Ohashi K., Sugimoto C., Onuma M. (1998). Pathogenesis of Marek’s Disease (MD) and Possible Mechanisms of Immunity Induced by MD Vaccine. J. Vet. Med. Sci..

[48] Islam T., Walkden Brown S.W., Renz K.G., Fakhrul Islam A.F.M., Ralapanawe S. (2013). Vaccination-Challenge Interval Markedly Influences Protection Provided by Rispens CVI988 Vaccine against Very Virulent Marek’s Disease Virus Challenge. Avian Pathol..

[49] Shek W.R., Calnek B.W., Schat K.A., Chen C.H. (1983). Characterization of Marek’s Disease Virus-Infected Lymphocytes: Discrimination between Cytolytically and Latently Infected Cells. J. Natl. Cancer Inst..

[50] Schat K.A., Chen C.-L.H., Shek W.R., Calnek B.W. (1982). Surface Antigens on Marek’s Disease Lymphoblastoid Tumor Cell Lines23. JNCIJ. Natl. Cancer Inst..

[51] Lee S.-I., Ohashi K., Morimura T., Sugimoto C., Onuma M. (1999). Re-Isolation of Marek’s Disease Virus from T Cell Subsets of Vaccinated and Non-Vaccinated Chickens. Arch. Virol..

[52] Schat K.A., Chen C.L., Calnek B.W., Char D. (1991). Transformation of T-Lymphocyte Subsets by Marek’s Disease Herpesvirus. J. Virol..

[53] Novy P., Quigley M., Huang X., Yang Y. (2007). CD4 T Cells Are Required for CD8 T Cell Survival during Both Primary and Memory Recall Responses. J. Immunol..

[54] Matsuyama-Kato A., Iseki H., Boodhoo N., Bavananthasivam J., Alqazlan N., Abdul-Careem M.F., Plattner B.L., Behboudi S., Sharif S. (2022). Phenotypic Characterization of Gamma Delta (Γδ) T Cells in Chickens Infected with or Vaccinated against Marek’s Disease Virus. Virology.

[55] Matsuyama-Kato A., Shojadoost B., Boodhoo N., Raj S., Alizadeh M., Fazel F., Fletcher C., Zheng J., Gupta B., Abdul-Careem M.F. (2023). Activated Chicken Gamma Delta T Cells Are Involved in Protective Immunity against Marek’s Disease. Viruses.

[56] Hao X., Li S., Li J., Yang Y., Qin A. (2021). An Anti-Tumor Vaccine Against Marek’s Disease Virus Induces Differential Activation and Memory Response of Γδ T Cells and CD8 T Cells in Chickens. Front. Immunol..

[57] Sabsabi M.A., Kheimar A., You Y., von La Roche D., Härtle S., Göbel T.W., von Heyl T., Schusser B., Kaufer B.B. (2024). Unraveling the Role of Γδ T Cells in the Pathogenesis of an Oncogenic Avian Herpesvirus. mBio.

[58] Schat K.A., Kaspers B., Kaiser P. (2014). Avian Immunology.

[59] Barrow A.D., Burgess S.C., Baigent S.J., Howes K., Nair V.K. (2003). Infection of Macrophages by a Lymphotropic Herpesvirus: A New Tropism for Marek’s Disease Virus. J. Gen. Virol..

[60] Djeraba A., Bernardet N., Dambrine G., Quéré P. (2000). Nitric Oxide Inhibits Marek’s Disease Virus Replication but Is Not the Single Decisive Factor in Interferon-γ-Mediated Viral Inhibition. Virology.

[61] Kodama H., Mikami T., Inoue M., Izawa H. (1979). Inhibitory Effects of Macrophages against Marek’s Disease Virus Plaque Formation in Chicken Kidney Cell Cultures. J. Natl. Cancer Inst..

[62] Chakraborty P., Kuo R., Vervelde L., Dutia B.M., Kaiser P., Smith J. (2019). Macrophages from Susceptible and Resistant Chicken Lines Have Different Transcriptomes Following Marek’s Disease Virus Infection. Genes.

[63] Mitchell R.A., Liao H., Chesney J., Fingerle-Rowson G., Baugh J., David J., Bucala R. (2002). Macrophage Migration Inhibitory Factor (MIF) Sustains Macrophage Proinflammatory Function by Inhibiting P53: Regulatory Role in the Innate Immune Response. Proc. Natl. Acad. Sci. USA.

[64] Zhu Z.J., Teng M., Liu Y., Chen F.J., Yao Y., Li E.Z., Luo J. (2024). Immune Escape of Avian Oncogenic Marek’s Disease Herpesvirus and Antagonistic Host Immune Responses. NPJ Vaccines.

[65] Hearn C., Preeyanon L., Hunt H.D., York I.A. (2015). An MHC Class I Immune Evasion Gene of Marek’s Disease Virus. Virology.

[66] Hunt H.D., Lupiani B., Miller M.M., Gimeno I., Lee L.F., Parcells M.S. (2001). Marek’s Disease Virus down-Regulates Surface Expression of MHC (B Complex) Class I (BF) Glycoproteins during Active but Not Latent Infection of Chicken Cells. Virology.

[67] Kim T., Hunt H.D., Parcells M.S., van Santen V., Ewald S.J. (2018). Two Class I Genes of the Chicken MHC Have Different Functions: BF1 Is Recognized by NK Cells While BF2 Is Recognized by CTLs. Immunogenetics.

