# Supplementary material for: Protection Conferred by Gallid Alphaherpesvirus 2 Vaccines Against Immunosuppression Induced by Very Virulent Plus (vv+) Marek’s Disease Virus Strains in Commercial Meat Type Chickens
Source: Pathogens. 2025 Jan 10;14(1):54. doi: 10.3390/pathogens14010054 (PMC11769226; doi:10.3390/pathogens14010054)
Supplement: Supplementary file 1 [file pathogens-14-00054-s001.zip › pathogens-3412939-supplementary.pdf]

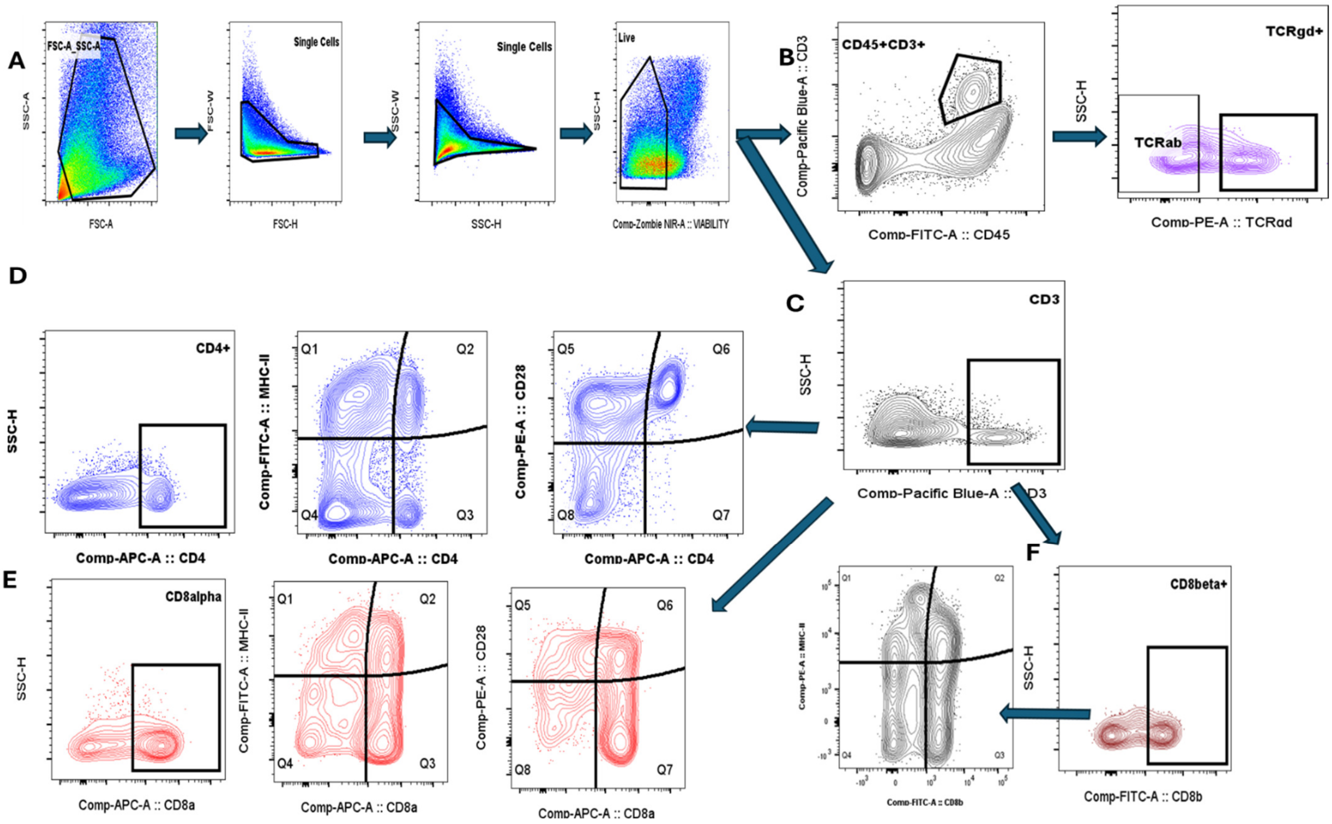

Figure S1. Gating strategy used For T cell phenotyping; Panel A: General gating strategy used for the analysis included gating on lymphocyte single cell splenocytes using forward (FSC) and Side (SSC) scatter parameters of width (W) and height (H) to exclude doublet cells followed by gating on the live cell population (A). To gate on γδ+ T cells (TCRgd), CD45+CD3+ double gating from live cells was conducted and then drilled down to gate on TCRγδ+ cells (B). In panel (C), from CD3+ T cells were gated from live cells gate and drilled down to obtain CD4+ cells, which was further drilled to CD4+CD28+ and CD4+MHC-II+ (D). The same for CD8α+ T cells, it was drilled from CD3+ T cell gate and further drilled into CD8α +CD28+ and CD8α +MHC-II+(E). Panel (F) shows the gating on CD8β+ T cells From CD3+ T cells then gating on CD8β+MHC-II+ T cells.

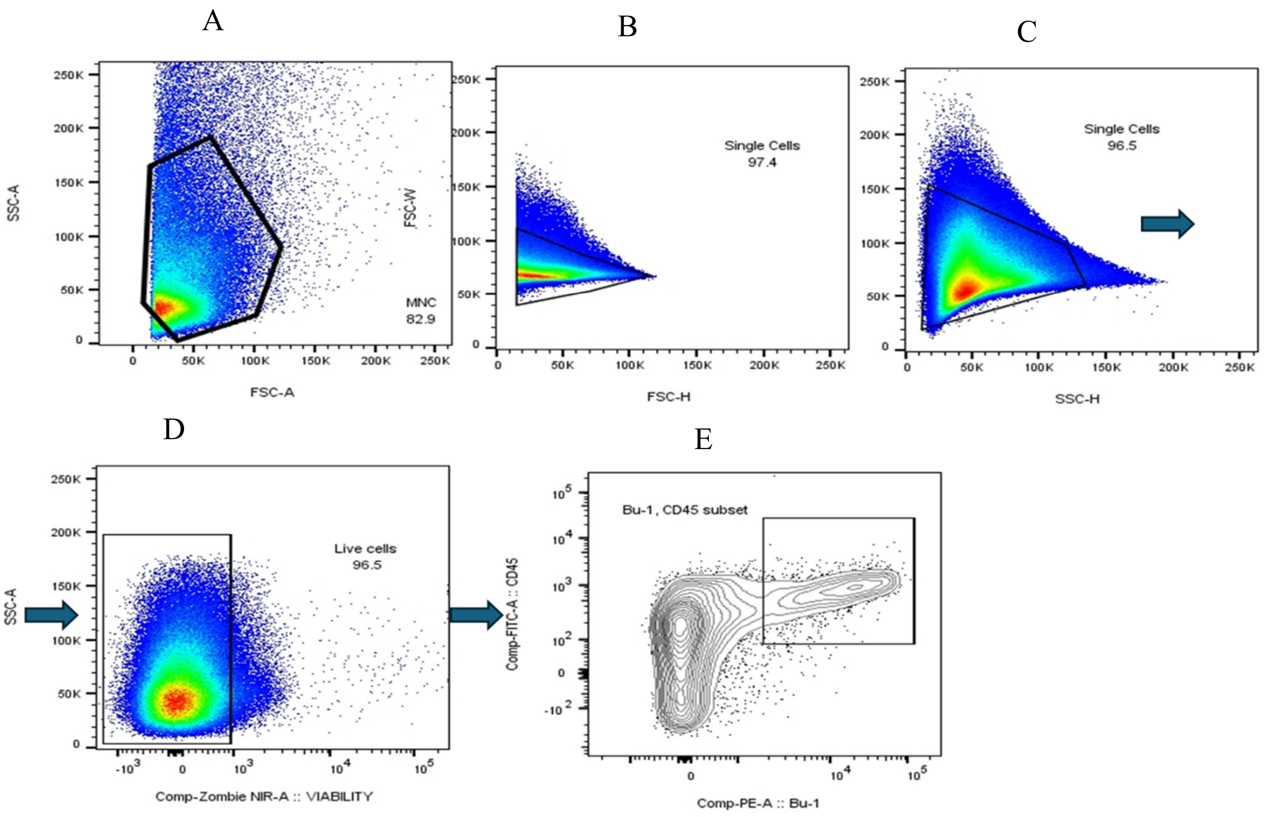

Figure S2. Gating on B cells: Gating strategy used included gating on lymphocyte single cell splenocytes using forward (FSC) and Side (SSC) scatter parameters of width (W) and height (H) to exclude doublet cells (A-C) followed by gating on the live cell population (D) followed by double gating on CD45+ Bu-1+ cells (Bu-1 is B cell marker antibody) (E).

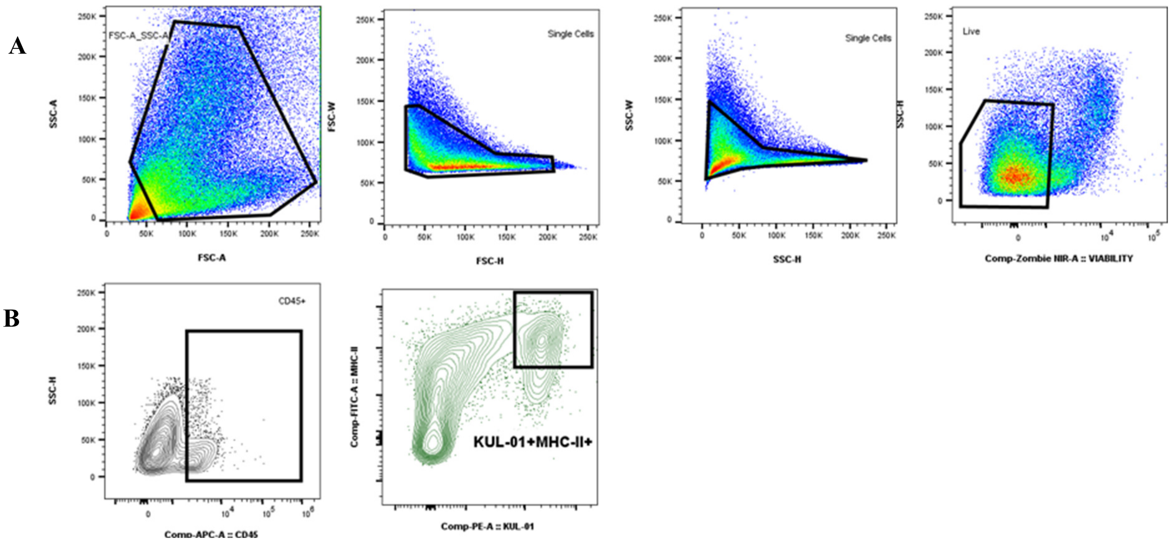

Figure S3. **Gating strategy used for activated macrophages**; Panel A: The gating strategy used for the analysis included gating lymphocyte single cell splenocytes using forward (FSC) and Side (SSC) scatter parameters of width (W) and height (H) to exclude doublet cells followed by gating on the live cell populations. Panel B: The live cells gate was drilled down to obtain and CD45+ cells followed by double gating on KUL-01 and MHC-II (activated macrophages).

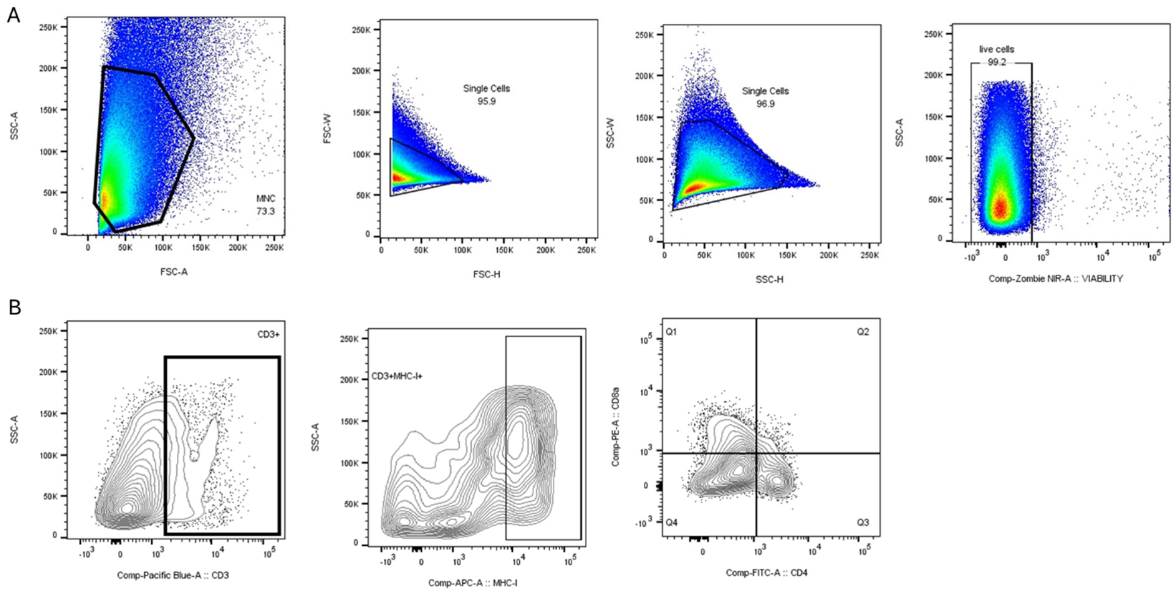

Figure S4. **Gating strategy used for MHC-I expression on different T cell subset phenotyping**; Panel **A**: The gating strategy used for the analysis included gating lymphocyte single cell splenocytes using forward (FSC) and Side (SSC) scatter parameters of width (W) and height (H) to exclude doublet cells followed by gating on the live cell populations. **Panel B**: The live cells gate was drilled down to obtain CD3+ MHC-I+ cells, which was further drilled to CD4+MHC-I+, CD8α+MHC-I+, CD4+CD8α+(DP) MHC-I+ and CD4-CD8α-(DN) MHC-I+ T cells after double gating on CD4+ and CD8+ T cells within MHC-I+ T cells.

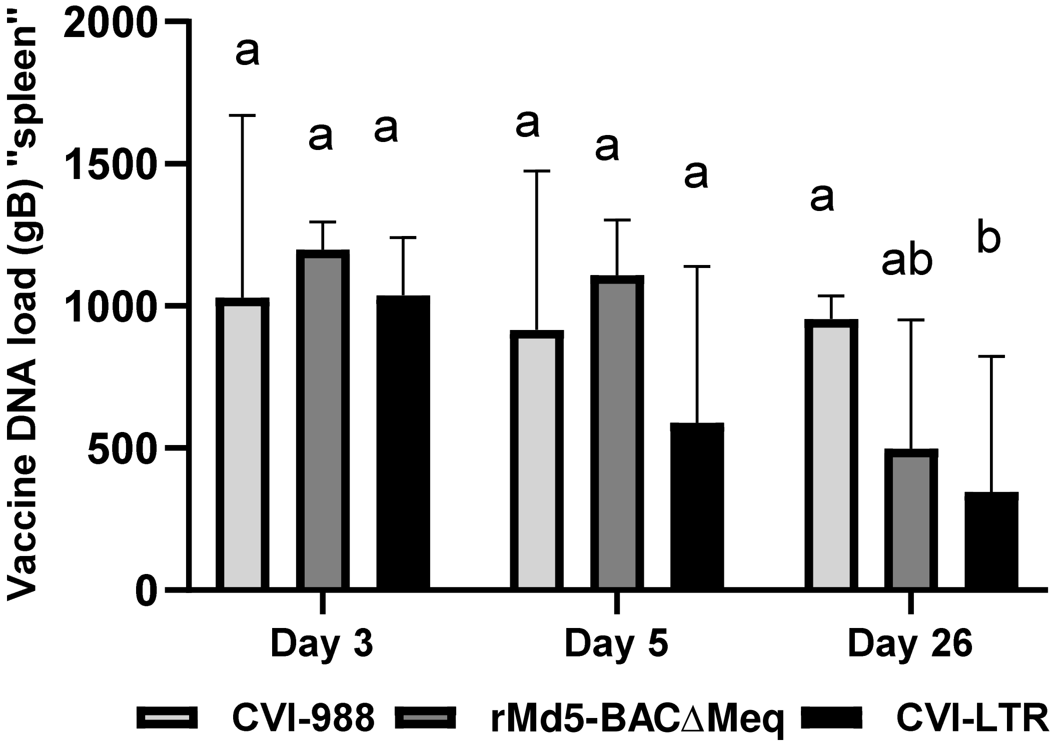

Figure S5. Replication (DNA load based on gB gene) of vaccines in the vaccinated unchallenged groups at 3, 5 and 26 days of age: Spleen samples were collected at indicated time points, DNA was extracted, and DNA samples were amplified with primers specific for the glycoprotein B (gB) gene of serotype 1 MDV. Viral DNA load was evaluated by the comparative Ct method (DDCT), accounting for primer efficiency, as described by Pfaffl (2001). Data was analyzed using 2 way ANOVA test followed by Fisher's LSD test for multiple comparisons. Different letters above the bars indicate that differences at were significant ($p \leq 0.05$).

Table S1. Staining panels for flow cytometry analysis of day 6[*]and 25 of age in meat type chickens.

Panel[1]	Antigen	Clone	Fluorochrome
	-	-	Live/Dead (L/D) Near Infra-red (IR)[2]
	CD3	CT-3	Pacific Blue (PB)™
1	CD4	CT-4	R-phycoerythrin (PE) + cyanine dye CY7(PE-Cy7)
	CD8α	CT-8	Allophycocyanin (APC)
	CD28	AV-7	Fluorescein Isothiocyanate (FITC)
	MHC-II	CIa	R-phycoerythrin (PE)
	-	-	Live/Dead (L/D) Near Infra-red (IR)
	CD3	CT-3	Pacific Blue (PB)™
2	CD8α	CT-8	Allophycocyanin (APC)
	CD8β	EP42	Fluorescein Isothiocyanate (FITC)
	MHC-II	CIa	R-phycoerythrin (PE)
	-	-	Live/Dead (L/D) Near Infra-red (IR)
	CD45	LT40	Fluorescein Isothiocyanate (FITC)
	CD3	CT-3	Pacific Blue (PB)™
3	CD4	CT-4	R-phycoerythrin (PE) + cyanine dye CY7(PE-Cy7)
	CD8α	CT-8	Allophycocyanin (APC)
	TCR-γ/δ	TCR 1	R-phycoerythrin (PE)
		-	Live/Dead (L/D) Near Infra-red (IR)
4	CD45	LT40	Fluorescein Isothiocyanate (FITC)
	Bu-1	AV20	R-phycoerythrin (PE)
	-	-	Live/Dead (L/D) Near Infra-red (IR)
	CD3	CT-3	Pacific Blue (PB)™
	CD4	CT-4	Fluorescein Isothiocyanate (FITC)
5	CD8α	CT-8	R-phycoerythrin (PE)
	MHC-I	C6B12[3]	Allophycocyanin (APC)
	-	-	Live/Dead (L/D) Near Infra-red (IR)
	CD45	LT40	Fluorescein Isothiocyanate (FITC)
6	Monocyte/macrophage	KUL01	Alexa Fluor® 647 (AF®6477)
	MHC-II	CIa	R-phycoerythrin (PE)

[1] 6 panels were used in this study: Panel 1, panel 2 and Panel 3 used antibodies, were directed against different T cell subsets including chicken T cell receptor (TCR) gamma delta (γδ) cells; Panel 4 used antibodies, directed against chicken B cell lymphocytes (Bu-1); Panel 5 used antibodies directed to detect MHC-I surface expression on different T cell subsets; Panel 6 used antibodies directed to detect macrophages.

[2] In all panels, exclusion of dead cells was performed by staining the cells with the Live/Dead™ Fixable Near-Infra-red (IR) Dead Cell Stain (Invitrogen, Carlsbad, CA).

3 the MCH-I colon C6B12 was obtained from the Developmental Studies Hybridoma Bank, University of Iowa (Iowa City, IA) and titrated to determine the optimal concentration for use and was detected by secondary anti mouse IgG on APC.

*At day 6 only panel 1, 2 and 3 and 6 were used due to unavailability of antibodies
